# Contrasting effects of the COVID-19 lockdown on urban birds’ reproductive success in two cities

**DOI:** 10.1038/s41598-021-96858-8

**Published:** 2021-09-03

**Authors:** Gábor Seress, Krisztina Sándor, Ernő Vincze, Ivett Pipoly, Boglárka Bukor, Nóra Ágh, András Liker

**Affiliations:** 1 MTA-PE Evolutionary Ecology Research Group, University of Pannonia, Veszprém, Hungary; 2 Behavioral Ecology Research Group, Center for Natural Sciences, University of Pannonia, 8200 Veszprém, Hungary

**Keywords:** Ecology, Urban ecology

## Abstract

The ubiquitous activity of humans is a fundamental feature of urban environments affecting local wildlife in several ways. Testing the influence of human disturbance would ideally need experimental approach, however, in cities, this is challenging at relevant spatial and temporal scales. Thus, to better understand the ecological effects of human activity, we exploited the opportunity that the city-wide lockdowns due to the COVID-19 pandemic provided during the spring of 2020. We assessed changes in reproductive success of great tits (*Parus major*) at two urban habitats affected strikingly differently by the ‘anthropause’, and at an unaffected forest site. Our results do not support that urban great tits benefited from reduced human mobility during the lockdown. First, at one of our urban sites, the strongly (− 44%) reduced human disturbance in 2020 (compared to a long-term reference period) did not increase birds’ reproductive output relative to the forest habitat where human disturbance was low in all years. Second, in the other urban habitat, recreational human activity considerably increased (+ 40%) during the lockdown and this was associated with strongly reduced nestling body size compared to the pre-COVID reference year. Analyses of other environmental factors (meteorological conditions, lockdown-induced changes in air pollution) suggest that these are not likely to explain our results. Our study supports that intensified human disturbance can have adverse fitness consequences in urban populations. It also highlights that a few months of ‘anthropause’ is not enough to counterweight the detrimental impacts of urbanization on local wildlife populations.

## Introduction

Urbanization often has negative effects on wildlife and the dramatic expansion of urban areas is a major factor responsible for the severe decline of animal populations worldwide^1–3^. One of the key features of urban environments is the ubiquitous presence and activity of humans that generate various stressors for urban wildlife, including chemical, acoustic, and light pollutions associated with vehicle traffic and direct disturbances caused by pedestrians and recreational activities in urban green spaces^4^.

To impede the spreading of the coronavirus disease (COVID-19), many countries went into lockdown during the spring of 2020. This resulted in greatly reduced human mobility and traffic in cities and towns across the world for several months, a phenomenon recently coined as ‘anthropause’^5^. Although observations suggest that urban wildlife quickly responded to the anthropause, for example by roaming in cities more freely than before^6,7^ or by adjusting their behavior to the new circumstances^8–11^, quantitative assessments of the ecological impacts in urban habitats are still rare.

In the northern temperate zone, the duration of the lockdown coincided with the breeding season of many animal populations, hence providing excellent opportunities to investigate the fitness consequences of human activities in urban wildlife populations. Under usual circumstances, these impacts are typically inferred from comparisons between populations living in habitats with different levels of urbanization or along urban-to-rural gradients^12^, because conducting experiments on relevant spatial and temporal scales in cities is challenging (but see^13^). Although some components of the urban environments can be effectively manipulated by experiments, e.g. food availability^14^, noise levels^15^, or light pollution^16^, other factors, such as road traffic and the ubiquitous presence of humans and the associated direct and indirect disturbances, are much more difficult to control in cities. For this reason, we have limited and mostly correlative evidence on the impacts of these anthropogenic effects on the fitness of urban animals.

Thus, in this study, we exploit the opportunity that the unprecedented conditions during the lockdown offered, and use a quasi-experimental approach to study the impacts of human activity on the breeding success of urban animals. To do so, we compare the reproductive performance of a widespread urban adapter bird, the great tit *(Parus major)* between areas where the lockdown induced markedly different effects on human activities. In a long-term study of this model system, we already showed that urban great tits have strongly reduced breeding success and nestling development relative to forest populations^14,17^. One main factor responsible for this disparity in breeding success is the limited availability of arthropod-rich nestling food (especially caterpillars) in urban areas^14,17^, although several other mechanisms may contribute to this phenomenon. For example, increased levels of traffic-related air pollution may affect urban birds detrimentally either via their health status^18^ or by reducing the abundance of their arthropod food supply^19^. Also, persistent exposure to anthropogenic noise may induce chronic stress^20^ and promote nest abandonment^21^, and can acoustically mask parent–offspring communication and other relevant auditory cues in birds’ environment resulting in decreased fitness^22^. Finally, high levels of human disturbance associated with, for example, human recreational activity or the increased presence of pets (e.g. dog walking) in urban green spaces may also disrupt parents’ nestling feeding patterns or constrain their access to foraging sites^23,24^.

**Figure 1:**
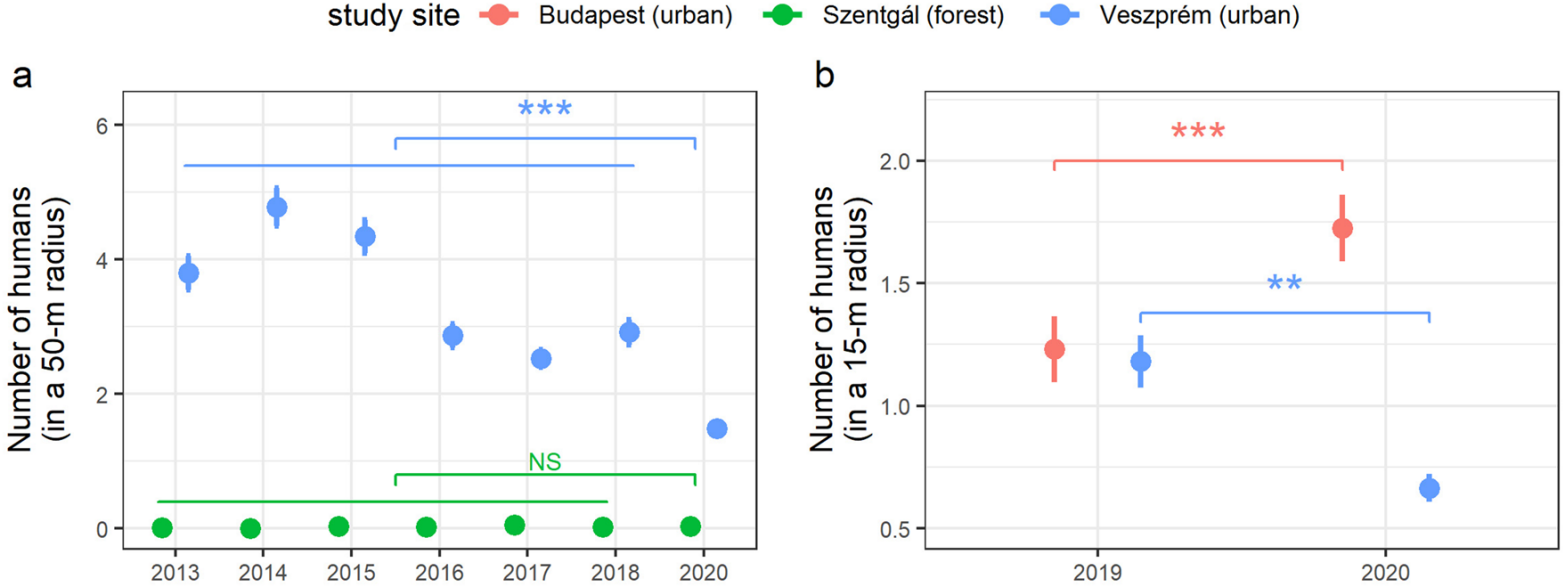
The number of humans (mean ± SE) recorded for 30-s counts in the proximity of active great tit nests during nest checks at (**a**) the forest (Szentgál) and urban (Veszprém) study sites in 2013–2018 (reference period) and 2020 and at (**b**) the two urban study sites (Budapest and Veszprém) in 2019–2020. Results are from linear models’ estimates (mean ± SE). Differences were statistically compared between 2020 (lockdown period) and the reference period: (**a**) 2013–2018 or (**b**) 2019 (***: 0 > *P* > 0.001; **: 0.001 > *P* > 0.01; *: 0.01 > *P* > 0.05), see main text for the results. The number of humans was recorded either within 50 m (**a**) or 15 m (**b**) (see Methods for explanation).

Therefore, we test the prediction that the large scale (city-wide) and persistent reduction in human activities due to the lockdown (in Veszprém, see below) during the birds’ breeding period should facilitate their reproductive success in urban areas^25^. To do so, first we compared the birds’ reproductive performance between a city (Veszprém) and a nearby mature forest area (Szentgál) that were affected differently by the movement restrictions during the spring of 2020, and compared this to 2019 as a reference (a year with no lockdown). While in 2020 the lockdown resulted in a significantly lower number of humans counted around the urban nests in Veszprém (compared to a long-term reference period), forest nests remained relatively undisturbed, similarly to the preceding years (Fig. 1a). Therefore, we predicted that the anthropause would yield positive effects on the birds’ reproductive success in the urban but not in the forest area, resulting in a reduced difference between the forest and the city in 2020 compared to their difference in 2019.

Second, we compared the birds’ reproductive success between two cities, Veszprém and Budapest (the capital city of Hungary), which responded in strikingly different ways to the lockdown. Interestingly, while the lockdown reduced human density in the public green areas of Veszprém by 44% (see above), we recorded a 40% increase in human presence around nest sites in the urban parks of Budapest, where residents used green spaces for recreational activities more frequently than in the preceding year (the levels of human presence were similar in the two urban areas in 2019; Fig. 1b). Building on this observation, we hypothesized that if human disturbance is indeed a major factor affecting reproductive success^26^, then birds should have a better breeding performance in Veszprém relative to Budapest in 2020 than in the pre-pandemic year (2019).

## Materials and methods

### Data collection

Data on the birds’ reproductive success and the number of humans present at nest sites were collected as part of a long-term, ongoing monitoring project in Hungary, in which we investigate the impacts of urbanization on populations of great tits. The great tit is an insectivorous passerine bird that is widespread across the Western Palearctic, occupies both urban and forest habitats, readily accepts nestboxes, and shares many important ecological traits with other tit or chickadee species also occurring in urban habitats^27^. These traits make this species an ideal model organism for studying the effects of the anthropause on wildlife in different environments.

#### Study sites

We monitored breeding great tit populations and also collected human presence data in two urban areas and at one forest study site. In one of the urban sites, Veszprém (47°05′17.29″N, 17°54′29.66″E; human population: c. 56,000; the monitoring scheme started in 2013), the nestboxes were placed in public green spaces (public parks, university campuses, a bus station, and a cemetery) that are surrounded by built-up areas and roads, and experience frequent anthropogenic disturbance. At the other urban size, Budapest (47°30′27.4"N, 19°01′03.4"E; the capital city of Hungary, human population: c. 1.75 million; the monitoring scheme started in 2019), the nestboxes were placed in two public urban parks, located c. 400 m from each other in the city core area and separated by high-traffic roads. The parks are freely accessible to residents and are heavily embedded within the urban matrix. At both urban sites, most of the nestboxes are distributed along paths or walking trails. Even though the two cities greatly differ in their size and human population, our urban study plots in both cities have similar general characteristics: these are surrounded by built-up areas, are at a similar distance (c. 3–4 km) from the nearest forested areas (for Veszprém, this is the forest at Vilma-puszta: 47°05′06.7″N, 17°51′51.4″E; for Budapest, this is the forest at Normafa: 47°30′27.7"N 18°57′51.1"E), and nests also experienced a similar level of human disturbance in the pre-COVID reference period (Fig. 1b). The forest site, Szentgál (47°06′39.75"N, 17°41′17.94"E; the monitoring scheme started in 2013), is a mature woodland, dominated by beech *(Fagus sylvatica)* and hornbeam *(Carpinus betulus)*, located 3 km away from the nearest human habitation (Szentgál, human population: c. 2.800), c. 20 km and 110 km away from Veszprém and Budapest, respectively. There are no paved roads in the forest, and the area is relatively free from human disturbance although it experiences occasional hunting and logging activity.

#### Human presence around nests

To quantify human presence at our study sites for 2020 and the reference years we counted the number of humans (motorized vehicles excluded) during each nest check, for 30 s, in the proximity of the nestboxes (for similar approach see Corsini et al. 2019). The number of humans was recorded within a 50-m radius of the nestboxes between 2013 and 2018 (Veszprém, Szentgál), and within a 15-m radius distance in 2019–2020 (all sites). We changed the counting distance in 2019 due to methodological reasons following^28^. However, to be able to compare the human presence data of 2020 in Veszprém and Szentgál to that recorded in earlier years, in 2020 we performed the counts with both the 15-m and the 50-m radius distances at these two sites. Thus, for 2020 in Veszprém, we have human presence data both for the 50-m and the 15-m radius areas that were used in the forest-city and the between-cities comparisons, respectively (see below). For each year and study site, we used human presence data only from seasonally first broods (defined below), and only from nests where there were either already eggs or nestlings in the nest, resulting in 9.4 ± 3.6 (mean ± SD) observations per brood which is a reliable indicator of human presence^28^.

#### Birds’ reproductive success

We monitored nestboxes each year at least twice a week from mid-March to early June to record laying and hatching dates, clutch size, hatching success, and the number of nestlings in active great tit nests. We ringed nestlings at day 14–16 post-hatch (i.e. a few days before fledging; hatching day of the first chick = day 1) with a numbered metal ring and also recorded their body mass (to the nearest 0.1 g), tarsus length (to the nearest 0.1 mm and following Svensson’s ‘alternative’ method^29^) and wing length (from the bend of the wing to the longest primary; to nearest 1 mm). Shortly after the expected date of fledging we carefully examined the nest material to identify and count the number of chicks that died after ringing (due to e.g. starvation, predation) that we included in the calculation of nestling survival (detailed below). The aim of this is to get a more accurate estimate for the number of offspring that could indeed fledge from the nest. The number of broods (nestlings) that suffered partial or complete mortality between ringing and fledging were: n = 6 (13) in Budapest (2019–2020), n = 70 (152) in Veszprém (2013–2020), and n = 25 (83) in the Szentgál forest.

From these data we determined clutch size (the maximum number of eggs observed in a brood), hatching success (the proportion of chicks hatched / eggs laid), the number of fledglings, and nestling survival (the proportion of fledged young / hatched chicks). The number of fledglings (i.e. the number of young fledged successfully) was calculated as the number of chicks ringed minus the number of chicks found dead in the nest after the ringing. We involved only seasonally first breeding attempts (as this period overlapped with the lockdown period; detailed at the Statistical analyses), and defined first broods as follows. In our study system breeding great tits are captured on their nests and receive a unique combination of colour rings. Active nests are also routinely equipped with a small, concealed video camera enabling us to reliably identify over 80% of breeding individuals each year^30^. Thus, relying on this setup, we considered a clutch as a first breeding attempt of a pair if it was initiated before the date of the first egg laid in the earliest second clutch at that site by an individually identifiable (i.e. colour-ringed) female that successfully raised her first clutch (i.e. fledged at least one young) in that year.

#### Air pollution and meteorological conditions

To describe the levels of traffic-related air pollution (nitrogen dioxide [NO_2_], nitrogen oxides [NO_X_] and ozone [O_3_]) and the meteorological conditions (temperature and precipitation) at the two urban study sites (Veszprém and Budapest), we used data provided by the Hungarian Air Quality Monitoring Network and the Hungarian Meteorological Service, respectively. To better understand which aspect of the anthropause might have affected great tits’ breeding success we thus assessed if the lockdown affected air pollution levels differently at the two urban study sites (compared to 2019), or if weather conditions showed different fluctuations between 2019 and 2020 at the two cities. For more details on the statistical analyses and results, see ESM: Sect. 1.

### Statistical analyses

The duration of the official restrictions on human mobility (lockdown) spanned between 28 March–4 May in Veszprém (calendar date: 88–125; 01 January = 1) and 28 March–18 May (88–139) in Budapest. During this period people were allowed to leave their homes e.g. to run essential errands including individual sport and recreational activities in public green spaces, although with keeping at least 1.5 m from each other (social distancing). Very importantly, from the point of view of our study, the period of movement restrictions had almost completely overlapped with the seasonally first breeding attempts (from egg-laying to fledging) of great tits at both urban sites. The date of laying the 1st egg (calendar date, mean ± SD) in Veszprém was 94.2 ± 6.4, while in Budapest 97 ± 7.8; the date of chick ringing and measuring in Veszprém was 128 ± 5.3, while in Budapest: 133 ± 9.1. Thus, we decided not to exclude any first broods based on the date in order to maximize our sample size. Similarly, the period from which we involved human presence data was also strongly overlapped with the duration of the movement restrictions in both cities. Therefore, in Veszprém, the calendar dates of the first and the last human count at each nest were 87–108 (median: 100) and 121–142 (median: 132), respectively, while in Budapest 87–128 (median: 98) and 118–155 (median: 128).

#### Human presence around nests

In accordance with our first objective (forest-city comparisons), we explored if the lockdown in 2020 caused any changes in human disturbance around the great tit nests. To do so, we compared the number of humans (50-m radius of the nests) between 2020 and the 2013–2018 reference period, separately for the forest (Szentgál) and urban (Veszprém) study sites. Note that in 2019, we did not collect data on human presence within a 50-m radius at Veszprém and Szentgál (see above: Data collection), therefore 2019 was not included in the reference period of this analysis. We, however, also compared human presence in Veszprém between 2019 and 2020 using the 15-m radius data which indicates a change that is consistent with the differences found using the 50-m radius data (detailed below).

First, we built generalized linear mixed-effects (GLM, *lme4* R package) models with Poisson error distribution with the number of humans as the response variable, including year as a fixed factor and nestbox ID as random factor to control for non-independence of the data. Next, we extracted the mean values (least-squares means; package *emmeans*^31^) and associated standard errors for each year as estimated by the model. We computed the mean of these yearly mean estimates for the 2013–2018 reference period (i.e. calculated a single overall mean describing the whole reference period) and compared this long-term mean to the mean estimate of 2020 by calculating the linear contrast between them (with the ‘contrast’ function of the *emmeans* package), and expressed linear contrasts as 2020 minus the reference period.

For our second objective (between-cities comparisons), we compared the changes in human disturbance around the nestboxes at the two urban study sites, Veszprém and Budapest, using the number of humans recorded within the 15-m radius of the active nests in 2019 and 2020. We analysed the data from Budapest and Veszprém separately and built generalized linear mixed-effects models with Poisson error distribution with the number of humans (15-m radius of the nests) as the response variable, including year as a fixed factor and nestbox ID as random factor to control for non-independence.

#### Birds’ reproductive success

We used data from 2019 (reference; for justification see below in this section) and 2020 (lockdown). First, we constructed separate linear models to analyse each component of reproductive success (response variables), and for the forest-city and the between-cities comparisons. We used linear models (LM) for clutch size and the number of fledglings, generalized linear models (GLM, with quasi-binomial error distribution) for hatching success and nestling survival, and linear-mixed effects models (LME) for nestling body size traits (body mass, tarsus length, and wing length). Models on nestling body size traits contained nestlings’ age at ringing as a confounding variable (three-level factor: 14, 15, or 16 d of age) and brood ID as a random factor to control for the non-independence of chicks raised in the same brood. Finally, these models always contained a habitat (Veszprém or Szentgál) × year (2019 or 2020) interaction term for forest-city comparisons and a city (Budapest or Veszprém) × year (2019 or 2020) interaction term for between-cities comparisons. We checked assumptions of residuals’ normality and homogeneity of variance by inspecting the residuals plots which were respected for all models.

Next, to test the prediction for our first objective (forest-city comparisons), we extracted the mean values (least-squares means) and associated standard errors of each response variable for each habitat × year combination as estimated by the linear model’s interaction. Then, from these estimates, we calculated habitat contrasts, i.e. the mean forest-city difference (forest minus urban) for each year (i.e. for 2019 and 2020), and compared the mean habitat contrast for the 2019 reference year to the mean habitat contrast of 2020; for similar approach see^14,32,33^.

For our second objective (between-cities comparisons), we followed the same procedure as for the forest-city comparisons (detailed above) except that here we compared the differences between cities (Budapest minus Veszprém) in 2020 and 2019. These full models (i.e. for the forest-city and between-cities comparisons) are presented in Table S1–S2 (ESM: Sect. 2).

In our study, we chose 2019 as a reference year for multiple reasons. First, because this was temporally the closest year without a lockdown. Second, because for Budapest we have monitoring data only from 2019 to 2020, using 2019 and 2020 in all analyses makes the results more comparable. Finally, although we have monitoring data from a total of eight years (2013–2020) for Szentgál (forest site) and Veszprém (urban site), for the forest-city comparisons we did not include years before 2019 in the reference because we noticed a negative trend in birds’ reproductive success throughout the study years (Fig. S3). This trend was especially apparent in the forest population, and may have reduced the forest-city difference by the end of the study period. Indeed, 2019 and 2020 were amongst the poorest years and resulted in a very similar reproductive success between both years within both habitats (Fig. S3). Because such temporal trend may have confounded the comparisons of 2020 with earlier years, to take account for its effect, and to further justify our approach of using 2019 as the reference year, we conducted additional analyses on the birds’ reproductive success by comparing both 2019 and 2020 (separately) to the 2013–2018 long-term reference period. We predict that if 2019 and 2020 are similarly affected by the decreasing trend in reproductive success than then the differences between the long-term reference period and 2019 and 2020, respectively, should be similar. For the details of these long-term forest-city comparisons see ESM: Sect. 3 and Table S3).

Finally, we did not conduct the forest-city comparisons (first objective) between the forest site (Szentgál) and the other urban site (Budapest) for two reasons. First, because unlike to the Szentgál vs. Veszprém setup, we did not have an appropriate forest (control) location which is close to Budapest. Second, because conducting comparisons between the long-term data and 2019 and 2020, respectively (see: ESM Sect. 3) was not possible for Budapest because we do not have similar long-term data for the latter site.

Clutches that failed before reaching the incubation stage (due to predation or desertion; i.e. final clutch size was uncertain), suffered complete mortality due to weather (e.g. nestbox fall from the tree due to strong wind), and cases when complete or partial clutch or brood loss may have occurred due to the monitoring process (e.g. when a nestbox was dropped or when complete brood failure occurred soon after capturing a parent on the nest) were excluded from all analyses. In the analyses investigating the number of fledglings, fledging success, and nestling body size traits we involved nests only where at least one nestling hatched, and excluded broods that were involved in a food-supplementation experiment (as treatment group) during the nestling rearing period in 2017^14^. We used the R 4.0.5 software environment for statistical analysis and creating figures^34^.

### Ethical statement

All procedures were in accordance with Hungarian laws, and adhered to the ASAB/ABS guidelines for the use of animals in behavioural research and teaching. Permit to the use of animals in this study was provided by the National Scientific Ethical Committee on Animal Experimentation (permit number: PE-06/KTF/997–8/2018, FPH061/1329–5/2018, PE-06/KTF/06,543–7/2020 and FPH061/3036–4/2020). Permits to study protected species and access to protected areas were provided by the Middle Transdanubian Inspectorate for Environmental Protection, Natural Protection and Water Management (permit numbers: 31559/2011, 24,861/2014 and VE-09Z/03,454–8/2018, for working in Veszprém and Szentgál) and the Environment Protection and Nature Conservation Department of the Pest County Bureau of the Hungarian Government and the Mayor's Office of Budapest (permit numbers: PE-06/KTF/997–8/2018, FPH061/1329–5/2018, PE-06/KTF/06,543–7/2020 and FPH061/3036–4/2020, for working in Budapest).

## Results

### Human presence around nests

Regarding our first objective (forest-city comparisons), our results indicated that, for Veszprém, the number of humans around the nests was significantly lower in 2020 than in the 2013–2018 reference period (linear contrast, mean ± SE = − 0.98 ± 0.06, z-ratio = − 17.132, *P* < 0.001, n = 2807 observations; Fig. 1a). In contrast, in Szentgál, we detected no such differences between 2020 and the previous years (linear contrast, mean ± SE = 0.52 ± 0.44, z-ratio = 1,167, *P* = 0.243, n = 2368 observations; Fig. 1a), as the number of humans registered within the 50-m radius of the nestboxes remained very low throughout the first-brood period similarly to the reference years (at Szentgál 98.4% (in 2020) and 99.0% (2013–2018) of the censuses indicated no humans around the nestboxes).

For our second objective (between-cities comparisons), we found that the number of humans around the nests in 2020 was significantly lower in Veszprém (b ± SE = − 0.48 ± 0.13, z-value = -3.761, *P* < 0.001, number of observations: 695) and significantly higher in Budapest (b ± SE = 0.42 ± 0.10, z-value = 4.103, *P* < 0.001, number of observations: 411) than in 2019 (Fig. 1b). Parameter estimates are expressed as 2020 compared to 2019, i.e. a negative value indicates a lower number of humans in 2020.

### Birds’ reproductive success

**Figure 2:**
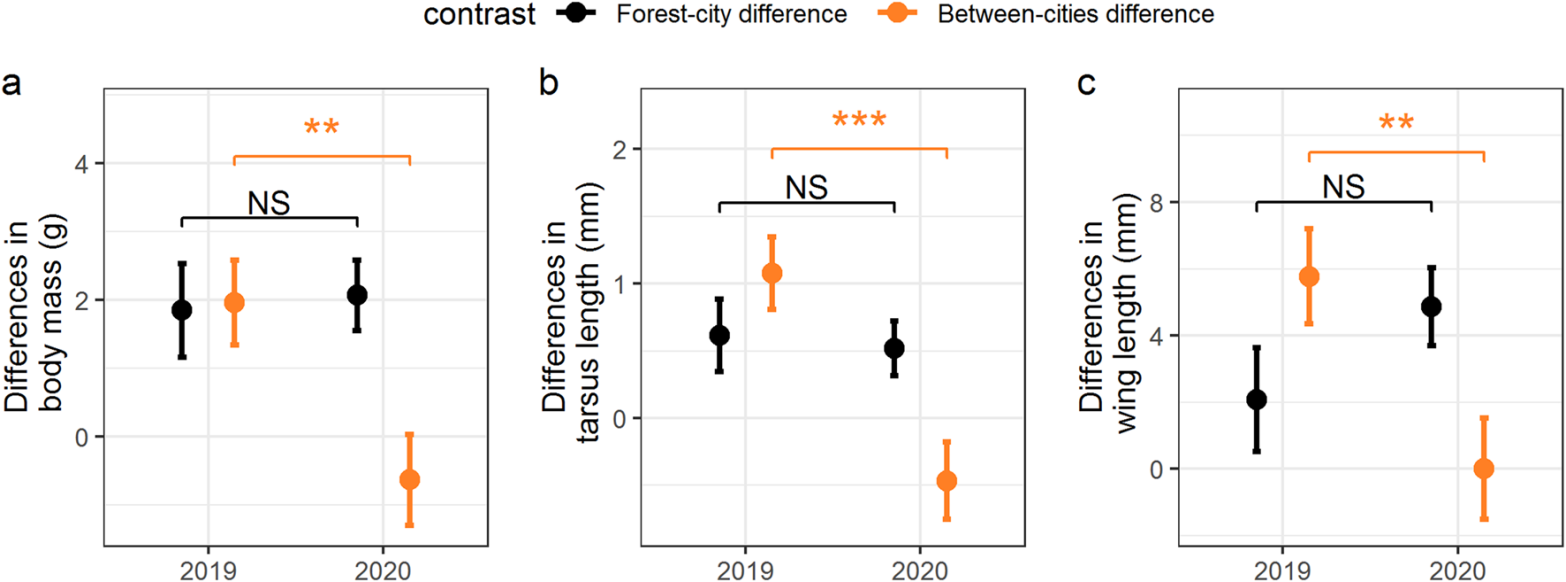
The impacts of the lockdown on nestlings’ body mass, tarsus length, and wing length in urban and forest populations of great tits. Black colour indicates comparisons between a forest (Szentgál, not affected by the lockdown) and a nearby urban site (Veszprém, where the lockdown decreased human activity around the nests), whereas orange colour shows comparisons between Veszprém and the capital city, Budapest (where the lockdown increased human activity at the study sites). Panels show the differences between these populations for 2020 (when breeding occurred during the lockdown) and for a reference year (2019). Differences are expressed as forest minus urban (forest-city comparisons) and Budapest minus Veszprém (between-cities comparisons), thus positive differences mean higher values in the forest or in Budapest, respectively, than in Veszprém. A smaller difference in 2020 than in 2019 indicates better relative performance of birds breeding in Veszprém during the lockdown. Number of broods (2019 and 2020), Szentgál: 11 and 22; Veszprém: 21 and 30; Budapest: 15 and 10. Results are from linear models’ estimates (mean ± SE). Population differences were statistically compared between 2019 and 2020 (***: 0 > *P* > 0.001; **: 0.001 > *P* > 0.01; *: 0.01 > *P* > 0.05), see Table 1 for details.

Regarding our first objective and contrary to our expectation, we found no significant changes from 2019 to 2020 in the forest-city differences in any components of great tits’ breeding success and nestling body size traits (Fig. 2; Table 1a), and the site × year interaction was also non-significant for any of the response variables (Table S1). Combined together, these results mean that the lockdown period in 2020 was not associated with an increased reproductive success in the city relative to the forest population. Our additional results for the forest-city comparisons using the long-term data (2019 and 2020 vs. 2013–2018) revealed that the forest-city differences in the birds’ reproductive success between 2019 and the long-term reference period was similar to those between 2020 and the long-term reference period (Table S3). This latter result indicates that (1) the long-term temporal trend in reproductive success is unlikely to be related to the lockdown-effect, and (2) justifies further the similarity between 2019 and 2020, despite that lockdown occurred only in 2020.

**Table 1:** Changes in population differences in the reproductive success of great tits between 2019 (reference period) and 2020 (breeding during the lockdown).

	Contrast ± SE	t or z ratio	*P*
**(a) Comparisons between forest and urban habitats: diff. in 2020 − diff. in 2019**			
Clutch size^1^	0.02 ± 0.45	0.05	0.963
Hatching success^2^	0.46 ± 0.63	0.73	0.465
Number of fledglings^3^	0.15 ± 1.03	0.15	0.883
Nestling survival^3^	0.01 ± 0.49	0.01	0.993
Nestling body mass (g)^4,5^	0.22 ± 0.85	0.26	0.793
Nestling tarsus length (mm)^4,5^	− 0.09 ± 0.34	− 0.29	0.771
Nestling wing length (mm)^4,5^	2.79 ± 1.95	1.43	0.156
**(b) Comparisons between cities (Budapest and Veszprém): diff. in 2020 − diff. in 2019**			
Clutch size^6^	− 0.09 ± 0.51	− 0.19	0.852
Hatching success^7^	− 1.03 ± 0.69	− 1.50	0.135
Number of fledglings^8^	− 1.16 ± 1.13	− 1.02	0.309
Nestling survival^8^	− 0.40 ± 0.63	− 0.63	0.529
**Nestling body mass (g)**^**4,9**^	− **2.59 ± 0.90**	− **2.89**	**0.005**
**Nestling tarsus length (mm)**^**4,9**^	− **1.54 ± 0.39**	− **3.98**	** < 0.001**
**Nestling wing length (mm)**^**4,9**^	− **5.76 ± 2.05**	− **2.82**	**0.006**

^1^Number of broods, total: 146; forest (Szentgál): 74, urban (Veszprém): 72.

^2^Number of broods, total: 139; forest (Szentgál): 68, urban (Veszprém): 71.

^3^Number of broods, total: 134; forest (Szentgál): 64, urban (Veszprém): 70.

^4^Results are averaged over the levels of nestlings’ age at ringing (three-level factor).

^5^Number of nestlings (broods), total: 488 (84); forest (Szentgál): 215 (33), urban (Veszprém): 273 (51).

^6^Number of broods, total: 112; urban (Budapest): 40, urban (Veszprém): 72.

^7^Number of broods, total: 107; urban (Budapest): 36, urban (Veszprém): 71.

^8^Number of broods, total: 99; urban (Budapest): 29, urban (Veszprém): 70.

^9^Number of nestlings and broods, total: 394 (76); urban (Budapest): 121 (25); urban (Veszprém): 273 (51).

Population differences were calculated either (**a**) between a forest and an urban habitat (Szentgál minus Veszprém) or (**b**) between two cities (Budapest minus Veszprém). Negative contrasts indicate smaller between-population differences during the 2020 lockdown than in 2019. Model estimated contrast ± SE values are on the original scales except for hatching success (the proportion of hatched chicks/laid eggs) and nestling survival (the proportion of fledged young/hatched chicks), which are given on the log odds ratio scale. Statistically significant results (*P* < 0.05) are highlighted in bold. See Table sS1 and S2 for statistical details of the models used for the forest-city and between-cities contrast calculations, respectively.

For our second objective, the between-cities comparisons, we found significant changes in the between-cities differences in nestling body size traits (as measured by body mass, tarsus length, and wing length) between 2019 and 2020 (Fig. 2a–c; Table 1b), in line with our prediction. These changes were driven by birds’ differing response to the lockdown in the two cities: while in Budapest nestlings reached significantly smaller body mass and size in 2020 than in 2019, in Veszprém we found no differences in nestlings’ body size traits between the two years (significant site × year interaction in all nestling traits, see Table S2. In contrast, we found no similar temporal changes in the between-cities differences for clutch size, hatching success, the number of fledglings, or nestling survival (Table 1b; non-significant site × year interactions, see Table S2).

## Discussion

Our results provide two key implications. First, as suggested by comparisons between a forest and a smaller city (Veszprém), contrary to our expectations, the lockdown did not eliminate urban birds’ disadvantage in reproductive success relative to a non-urban population, despite the considerably reduced human activities in the city during the breeding season of 2020. In theory, there are several possible explanations for this lack of effect. For example, it might be related to the relatively low levels of pre-lockdown human disturbance (traffic intensity, pedestrian density) in smaller cities like Veszprém. However, this explanation is not likely, as in 2019 the number of humans recorded around the nests in Veszprém was very similar to that recorded in the urban parks of Budapest (Fig. 1b), although the behavior and activity of people (hence the intensity of disturbance) may differ between the two cities. Alternatively, the prolonged reduction in human presence could also have led to an increase in the predator numbers in the urban parks of Veszprém^6,35^, resulting in increased nest predation and therefore counterweighing the putative positive effects of the lockdown. However, this explanation is also unlikely, as in 2020 we did not register any nest predation events (evidenced by the complete disappearance of the whole brood or by the predator marks on the nest structure) in the urban nests included in the study. An alternative and in our opinion more likely explanation for the lack of lockdown-effect in Veszprém is that the difference in reproductive success between the city and the nearby forest site (Szentgál) is mainly driven by differences in food availability with relatively low contribution from other anthropogenic effects. This interpretation is strongly supported by the considerably lower abundance of caterpillars (the principal nestling food for great tits) at our urban compared to forest sites^17^, and especially by a food supplementation experiment that eliminated much of the forest-city differences in both nestling body size traits and nestling survival^14^.

Second, nestlings’ body size traits were strongly reduced during the lockdown in the parks of Budapest where recreational activities significantly increased human presence (hence probably the intensity of disturbance to birds) in the proximity of nests compared to 2019. Importantly, this hindered nestling development might have posed fitness consequences for birds in Budapest, as reduced pre-fledging body mass predicts lower first-year survival in many songbird species including great tits^36^. These results provide one of the rare large-scale quasi-experimental supports for the hypothesis that human disturbance can pose considerable fitness costs for animals even at habitats where high level of human presence is common, and even for such a successful urban-dweller species like the great tit. The negative impacts on nestlings’ body size traits may be caused by the adverse effects of increased disturbance in several, non-exclusive ways. Human disturbance can act as a stressor resulting in elevated stress hormone levels (e.g. glucocorticoid concentrations) in animals, helping them to cope with stressful conditions at the cost of suppressing other activities like reproduction or parental care^37^ as it was demonstrated in other urban-breeding bird species^21^. Similarly, increased human activity and its by-products (e.g. noise) can also have subtle impacts on birds’ behaviour via energetic costs (due to more frequent fleeing or the maintenance of increased vigilance state) or by lost opportunities^38^. For example, human disturbance can disrupt birds’ foraging patterns, hindering parent–offspring communication^22^, constraining birds’ access to preferred foraging sites^23^, increasing birds’ stress levels^20^, or reducing parents’ foraging success, which has also been shown in other species^24,39^. Further, with the increased human presence in Budapest, not only human disturbance but also the presence of pet animals such as dogs could have increased during the lockdown. Contrary to this assumption, our regular predator surveys indicated similar ‘predator’ numbers (mostly dogs) in the proximity of the nestboxes in 2019 and 2020 (mean predator number was 2.28 in 2019 and 1.91 in 2020; Wilcoxon-test: W = 22,305, *P* = 0.313; Vincze et al., unpublished data). Additionally, in the downtown parks of Budapest (where our study sites are located), dog walking is only allowed on a leash, therefore we argue that domestic dogs pose a very low predation risk to adult great tits—although their mere presence can evoke non-lethal effects in birds^40^ thus contributing to the decreased nestling body size traits we registered during the lockdown. All these above-mentioned impacts can be especially strong in food-limited environments. In line with this idea, a recent meta-analysis found a massive decline in the abundance and diversity of arthropods, especially for lepidopterans, in temperate zone cities^41^. Under such conditions, a sudden and lasting increase in another stressor (e.g. human disturbance) may further limit the birds’ feeding success and hinder the already impaired nestling development. Note that the opposite may not be necessarily true—as illustrated by that great tits at Veszprém could not realize higher breeding success in 2020 (compared to earlier years) under significantly reduced human disturbance around their nests. Finally, we found lower clutch size in Budapest compared to Veszprém in both years (by 1.4 eggs in 2019 and 1.5 eggs in 2020; Fig S3) although we argue that this is unlikely to generate the changes we detected in the between-cities differences in nestling size. Instead, the lower clutch size could have made it even easier for parents in Budapest to mitigate the effect of extra disturbance in 2020 due to that parents needed to provision a smaller number of nestlings. Therefore, a smaller clutch/brood size could have potentially reduced the chance to detect an effect of disturbance on reproductive parameters, making our study more conservative.

In contrast with nestlings’ body size, we found no temporal changes in the between-cities differences for clutch size, hatching success, the number of fledglings, or nestling survival. These latter results might be explained, at least to some extent, by the relatively short period elapsed from the beginning of the lockdown to the initiation of the egg-laying (on average c. 6 days (Veszprém) and 9 days (Budapest); see Methods), because in this way birds did not experience the ‘lockdown effect’ during most of their pre-laying period (March). However, conditions during the pre-breeding stage (e.g. females’ physiological state during egg formation) are also known to affect parental care or reproductive success later in the breeding season. For example, a study on house sparrows found that females with high baseline corticosterone (the main glucocorticoid hormone in birds) levels c. 3–4 weeks before egg-laying produced lower number of fledglings during the breeding season^42^. Therefore, such pre-breeding effects might have reduced the potential impacts of the changes in human disturbance on birds’ clutch size (and perhaps also on fledgling number).

Besides inducing changes in human activity, the lockdown had other consequences that could have affected urban birds’ breeding by different means, although we argue that these may be less likely in our case. For example, the reduction in vehicular road traffic during the lockdown was paralleled by reduced concentrations of air pollutants, resulting in improved air quality worldwide^43^ that could have beneficial effects on urban birds directly e.g. by affecting their oxidative stress physiology^18^ or indirectly (via their arthropod food supply). However, while in Budapest the levels of NO_2_ and NO_X_ (two reliable indicators of traffic-related urban air pollution, measured during birds’ first-brood period) were substantially reduced during the lockdown compared to 2019 (Fig. S1) or longer reference periods^43^, in Veszprém the levels of both pollutants were similar in 2019 and 2020 (Fig. S1). Note that these different changes in air pollution in the two cities would predict an opposite change in birds’ reproductive performance of what we detected in our urban populations. Decreased vehicle traffic can, however, result in actually elevated levels of another air pollutant, the ground-level O_3_. Indeed, O_3_ concentrations were found to be strongly increased in several cities during the lockdown due to the unprecedented reductions of NO_X_ emissions^44^. High levels of O_3_ have several harmful impacts on birds’ reproductive success, and a recent long-term, continental-scale study demonstrated negative associations between bird abundance and O_3_ concentrations in the United States^45^. Even though in our study system the levels of O_3_ were higher in Veszprém than in Budapest (both in 2019 and 2020), its concentrations did not change significantly in either city during the lockdown when compared to the 2019 reference period (Fig. S1). Therefore, building on these results, we conclude that it is not likely that the lockdown-associated changes in the levels of the studied air pollutants can explain the differences in birds’ reproductive success between the cities. Finally, we can also exclude that differences in meteorological conditions at our two urban study sites biased our results for two reasons. First, both daily temperature and the amount of daily precipitation (measured during birds’ breeding period) changed similarly at the two urban study sites between 2019 and 2020 (ESM: Sect. 1.2 and Fig. S2). Second, the daily mean temperature in Budapest was very similar in 2019 (mean ± SE: 10.56 ± 0.43 C°) to that of 2020 (10.39 ± 0.43 C°), and the difference between Budapest and Veszprém was also similar in the two years (2019: 1.46 ± 0.6 C°; 2020: 0.82 ± 0.61 C°). Therefore, if the typical temperature difference between the two cities had played a major role in shaping our nestling body size results then in Budapest we would expect similarly low nestling body size traits in 2019 as in 2020—but this is not what our data shows.

In conclusion, our study demonstrates that reductions in human presence during the lockdown had little effect on great tits’ reproductive performance in urban habitats, where other factors, e.g. food availability, are known to strongly limit the birds’ reproduction. On the other hand, our study also showed that the lockdown could result in strongly increased human presence in some urban areas to a level that has potential detrimental fitness consequences for urban-breeding birds, even if the lockdown otherwise considerably reduced other negative anthropogenic effects like air and noise pollution. Taken together, these results suggest that, in ecological systems like ours, it would need more than just a few months of ‘anthropause’ to offset the detrimental impacts of urbanization, as the positive impacts resulting from the reduced human mobility very likely need more time to build-up and take effect on urban wildlife.

## Supplementary Information

Supplementary Information.

## Data Availability

The datasets used and/or analysed during the current study are available from the corresponding author on reasonable request.
