# Supplementary material for: Contrasting effects of the COVID-19 lockdown on urban birds’ reproductive success in two cities
Source: Sci Rep. 2021 Sep 3;11:17649. doi: 10.1038/s41598-021-96858-8 (PMC8417259; doi:10.1038/s41598-021-96858-8)
Supplement: Supplementary file 1 — Supplementary Information. [file 41598_2021_96858_MOESM1_ESM.docx]

**ELECTRONIC SUPPLEMENTARY MATERIAL**

**Contrasting effects of the COVID-19 lockdown on urban birds’ reproductive success in two cities**

G. Seress, K. Sándor, E. Vincze, I. Pipoly, B. Bukor, N. Ágh, A. Liker

**1. Environmental background data**

**1.1. Air pollution levels at the two urban study sites (Budapest and Veszprém, 2019-2020)**

Air pollution data for the two urban study sites, Budapest and Veszprém, were available from the Hungarian Air Quality Monitoring Network (<http://levegominoseg.hu/>; Hungarian Ministry of Agriculture). This network consists of automatic monitoring stations with continuous measurements of a wide range of air pollutants in ambient air. In both cities, we used the data from the monitoring stations that were the closest to our study sites (in Budapest: 47°30'31.284″N, 19°1'39.8352″E; distance c. 800 m; in Veszprém: 47°5'22.455″N, 17°54'17.9562″E; distance: c. 250 m). To describe air pollution resulting from vehicle emissions we used average daily concentrations of nitrogen dioxide (NO_2_), nitrogen oxides (NO_X_) and tropospheric ozone (O_3_). Other traffic-related pollutants were highly correlated with these thus were not considered further. These pollutants are potent oxidative stressors that are released into the air during the incomplete combustion of fossil fuels (Salma et al. 2020), and their levels are known to be highly correlated with the degree of urbanization (Salmón et al. 2018). Both NO_2_ and NO_X_ are often used as proxies for traffic-related air pollution in avian urban ecological studies (Peach et al. 2008, Bailly et al. 2017, Salmón et al. 2018) and also have negative impacts on the diversity and abundance of urban arthropods (Jones and Leather 2012, Fenoglio et al. 2020), resulting in potential further, indirect detrimental effects on urban insectivorous birds through their food supply. Similarly, high levels of tropospheric ozone in cities is considered to be a major hazard to human health (Sicard et al. 2018), and is also associated with severe declines in bird populations (Liang et al. 2020). During the 2020 lockdown, ozone concentrations showed strongly rising levels in cities worldwide as an indirect consequence of reduced emissions resulting from vehicle traffic (Sicard et al. 2020).

We used NO_2_, NO_X_ and O_3_ data covering only the complete first-brood period defined for each site and year separately as from the laying date of the first egg until the date at which the last nestling of the first broods was ringed. These periods were as follows: in 2019, Veszprém: 21 March–20 May (calendar date: 80–140; 01. Jan = 1) and Budapest: 26 March–20 May (calendar date: 85–140); in 2020, Veszprém: 21 March–18 May (calendar date: 81–139) and Budapest: 23 March–03 June (calendar date: 83–155).

We fitted separate linear models for the average daily concentrations of NO_2_ (Fig. S1ab), NO_X_ and O_3_ (response variables) that contained the city (Budapest or Veszprém) × year (2019-2020, as a two-level factor) interaction term. We also extracted the mean values and associated standard errors of each response variable for each city × year combination as estimated by the linear model’s interaction, and compared 2020 to 2019 (separately for each city) by calculating linear contrasts of these least squares means (package *emmeans*). Linear contrasts are expressed as 2020 minus the reference year (2019), i.e. negative values indicate lower pollution levels in 2020 than in 2019.

The city × year interaction proved to be highly significant for both NO_2_ and NO_X_, revealing that the level of these pollutants changed differently between 2019 and 2020 at the two urban study sites (NO_2_: b ± SE= 12.94 ± 2.83 µg/m^3^, t-value= 4.568, *P<* 0.001; NO_X_: 44.94 ± 5.09 µg/m^3^, t-value= 8.744, *P<* 0.001). The within-site comparisons also indicated that while in Veszprém both NO_2_ and NO_X_ levels were similar in 2019 and 2020 (NO_2_: contrast ± SE= -0.07 ± 1.03 µg/m^3^, t-ratio= -0.069, *P*= 0.945; NO_X_: -2.45 ± 1.85 µg/m^3^, t-ratio= -1.327, *P*= 0.186), in Budapest, the levels of both pollutants reduced significantly in 2020 (i.e. during the anthropause) compared to 2019 (NO_2_: contrast ± SE= -6.54 ± 0.98 µg/m^3^, t-ratio= -6.703, *P<* 0.001; NO_X_: -24.7 ± 1.76 µg/m^3^, t-ratio= -14.088, *P<* 0.001). For O_3,_ the city × year interaction was not significant (-0.05 ± 3.39 µg/m^3^, t-value= -0.014, *P*= 0.989) and the within-site comparison showed no difference between the two years as well (Veszprém: 1.66 ± 1.20 µg/m^3^, t-ratio= 1.376, *P*= 0.170; Budapest: 1.68 ± 1.19 µg/m^3^, t-ratio= 1.408, *P*= 0.161). The only remarkable difference we found between the two cities is that Veszprém had higher concentrations of O_3_ in both years than Budapest (Fig. S1c).

**Fig. S1.** The levels of traffic-related air pollution, **(a)** nitrogen dioxide (NO_2_), **(b)** nitrogen oxides NO_X_), and **(c)** ozone (O_3_) in 2020 (during the lockdown) and 2019 (reference year) during great tits’ breeding season (covering only the first-brood period) at the two urban study sites, Budapest and Veszprém. Air pollution data were recorded at monitoring stations (Hungarian Air Quality Monitoring Network) located close to our urban study sites. See the text for statistical comparisons and more details. On the graph medians and interquartile ranges are respectively indicated by the thick middle lines and the boxes, while the whiskers extend to the most extreme data points within 1.5 × interquartile range from the box, and the points refer to the outliers.


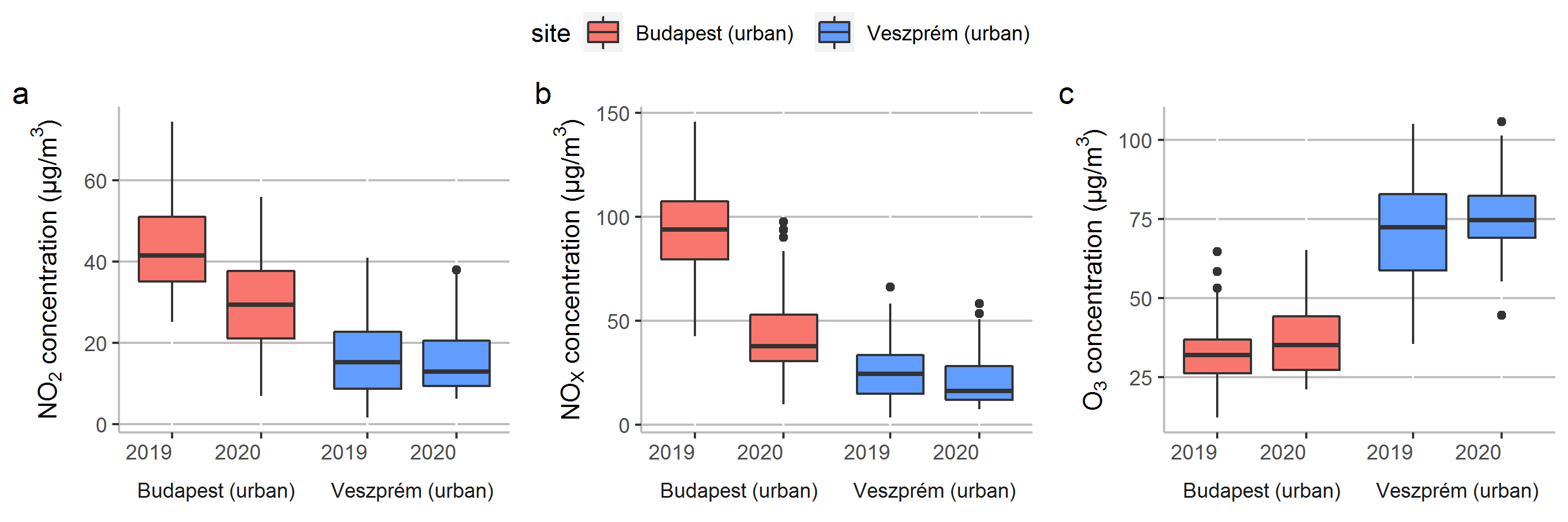


**1.2. Meteorological conditions at the two urban study sites (Budapest and Veszprém)**

To describe the meteorological conditions in our geographic area during the study, we used datasets from weather stations maintained by the Hungarian Meteorological Service, available in the NOAA database ([www.ncdc.noaa.gov](http://www.ncdc.noaa.gov)). For the Veszprém (urban) study site, we used the weather station data of Szentkirályszabadja, a village (47°03’21.07"N, 17°58'11.80"E) c. 6 km from Veszprém and 22 km from Szentgál; whereas, for the Budapest (urban) study site, we used the weather station data of Pestszentlőrinc, located within Budapest (47°25′50″N, 19°10′52″E), 14 km from our urban study sites. The weather stations recorded every three hours a day (0, 3, 6, 12, 15, 18, 21 UTC). From this data, we calculated the daily mean temperatures and daily amount of precipitation between 1 February and 31 May for the study years (2013-2020). This period covers the pre-breeding season (e.g. breeding site selection, territory occupation, and nest building) and also the complete first-brood period (from egg-laying to ringing and measuring of nestlings) of great tits in our geographic region.

To compare the changes in meteorological conditions between the two urban study sites in 2019-2020, we fitted separate linear models for daily mean temperatures and for the daily amount of precipitation (response variables). Both models contained the city (Budapest or Veszprém) × year (2019-2020, as a two-level factor) interaction term. Because the precipitation values were not linearly distributed, we used Generalized Estimating Equations models (GEE; *geepack* R package; Halekoh et al. 2006) to analyse the daily amount of precipitation that also contained calendar days as IDs.

The results indicated no significant city × year interaction either for the daily mean temperatures (b ± SE= 0.64 ± 0.86 ℃, t-value= 0.74, *P* = 0.458) or for the amounts of daily precipitation (b ± SE= 0.74 ± 0.85 mm, z-value= 0.77, *P* = 0.380), indicating that temperature and rainfall changed similarly at the two urban study sites between 2019 and 2020 (Fig S2).

**Fig. S2.** Mean ± SE of **(a)** daily mean temperatures and **(b)** daily amounts of precipitation between 1 February and 31 May in the study region for the eight study years (2013-2020). All temperature data were recorded at a meteorological station at Szentkirályszabadja (for study site at Veszprém, indicated by blue) and Pestszentlőrinc (for study site at Budapest, indicated by red). Differences were statistically compared between 2019 and 2020, see the text for the results.


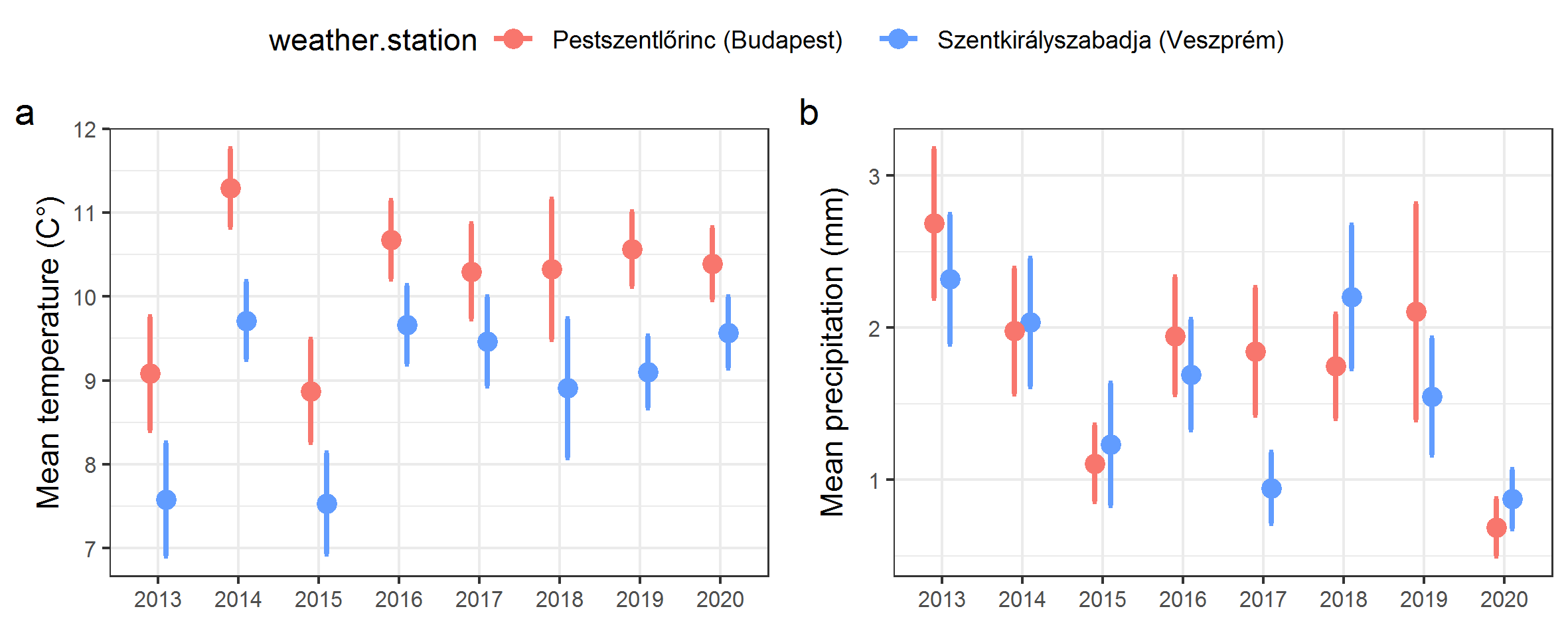


**2. Birds’ reproductive success: additional analyses and results for the forest-city (Szentgál vs. Veszprém) and the between-cities (Budapest vs. Veszprém) comparisons (2019-2020)**

Here we report the results of the models that were used to calculate and compare the forest-city (Szentgál vs. Veszprém) and the between-cities (Budapest vs. Veszprém) contrasts in birds’ breeding success for 2019 (reference year) and 2020 (lockdown), as reported in Table 1 (see Methods in the main text for further details on these calculations).

For the forest-city comparisons, the site (Szentgál or Veszprém) × year (2019 or 2020) interaction was not significant for any components of birds’ reproductive success (Table S1). For the between-cities comparisons, however, the site (Budapest or Veszprém) × year (2019 or 2020) interaction was highly significant for all nestling body size traits, but not for clutch size, hatching success, number of fledglings, and nestling survival (Table S2). These findings support the results of the linear contrast analyses (presented in Table 1 of the main text), i.e. that (1) the differences between the forest (Szentgál) and the urban (Veszprém) bird populations did not change between 2019 and 2020 (with effective lockdown in the urban study site), and also that (2) compared to 2019 birds at Veszprém (with the effective lockdown) performed relatively better than birds at Budapest (where human presence increased during the lockdown).

**Table S1.** The impacts of the anthropause on great tits’ reproductive success in a forest (Szentgál) and in an urban (Veszprém) habitat that were affected differently by the lockdown in 2020 (Veszprém: reduced human activity compared to earlier years, Szentgál: low human activity, no changes compared to earlier years, see Fig. 1a of the main text). The reference year is 2019. The table shows the results of the full statistical models that were used for calculating the contrasts presented in Table 1a of the main text. The forest site (Szentgál) and 2020 were set in the intercept. Results of hatching success (the proportion of hatched chicks/laid eggs) and nestling survival (the proportion of fledged young/hatched chicks) are given on the logit scale.

| **Forest-city comparisons (2019 and 2020)** | | b ± SE | t-value | *P* |
| --- | --- | --- | --- | --- |
| *Clutch size ^1^* | Intercept | 10 ± 0.25 | 40.57 | < 0.001 |
|  | Year (2019) | 0.32 ± 0.32 | 1.00 | 0.321 |
|  | Site (Veszprém) | -1.57 ± 0.33 | -4.73 | < 0.001 |
|  | Site × Year | 0.02 ± 0.45 | 0.05 | 0.963 |
| *Hatching success ^2^* | Intercept | 3.02 ± 0.44 | 6.81 | < 0.001 |
|  | Year (2019) | -0.79 ± 0.50 | -1.55 | 1.123 |
|  | Site (Veszprém) | -0.88 ± 0.53 | -1.68 | 0.095 |
|  | Site × Year | 0.46 ± 0.63 | 0.73 | 0.466 |
| *Number of fledglings ^3^* | Intercept | 5.00 ± 0.56 | 8.94 | < 0.001 |
|  | Year (2019) | -0.92 ± 0.75 | -1.23 | 0.221 |
|  | Site (Veszprém) | -1.00 ± 0.75 | -1.34 | 0.182 |
|  | Site × Year | 0.15 ± 1.03 | 0.15 | 0.883 |
| *Nestling survival ^3^* | Intercept | 0.09 ± 0.25 | 0.35 | 0.725 |
|  | Year (2019) | -0.37 ± 0.34 | -1.08 | 0.283 |
|  | Site (Veszprém) | -0.01 ± 0.36 | -0.03 | 0.977 |
|  | Site × Year | 0.00 ± 0.50 | 0.01 | 0.993 |
| *Nestling body mass (g) ^4^* | Intercept | 15.47 ± 0.46 | 33.94 | < 0.001 |
|  | Year (2019) | -0.15 ± 0.67 | -0.23 | 0.822 |
|  | Site (Veszprém) | -2.07 ± 0.51 | -4.04 | < 0.001 |
|  | Nestling age (day 15) ^5^ | 0.77 ± 0.46 | 1.68 | 0.096 |
|  | Nestling age (day 16) ^5^ | 0.54 ± 0.54 | 1.01 | 0.316 |
|  | Site × Year | 0.23 ± 0.85 | 0.26 | 0.793 |
| *Nestling tarsus length (mm) ^4^* | Intercept | 19 ± 0.18 | 106.32 | < 0.001 |
|  | Year (2019) | -0.11 ± 0.26 | -0.41 | 0.686 |
|  | Site (Veszprém) | -0.52 ± 0.2 | -2.56 | 0.012 |
|  | Nestling age (day 15) ^6^ | 0.16 ± 0.18 | 0.89 | 0.375 |
|  | Nestling age (day 16) ^6^ | 0.28 ± 0.21 | 1.32 | 0.191 |
|  | Site × Year | -0.1 ± 0.34 | -0.29 | 0.771 |
| *Nestling wing length (mm) ^4^* | Intercept | 42.87 ± 1.04 | 41.26 | < 0.001 |
|  | Year (2019) | -2.62 ± 1.54 | -1.71 | 0.092 |
|  | Site (Veszprém) | -4.87 ± 1.17 | -4.17 | < 0.001 |
|  | Nestling age (day 15) ^7^ | 3.13 ± 1.04 | 3.01 | 0.004 |
|  | Nestling age (day 16) ^7^ | 5.99 ± 1.22 | 4.92 | < 0.001 |
|  | Site × Year | 2.79 ± 1.95 | 1.43 | 0.156 |

^1^ Number of broods: total: 146; forest (Szentgál): 74, urban (Veszprém): 72

^2^ Number of broods, total: 139; forest (Szentgál): 68, urban (Veszprém): 71

^3^ Number of broods: total: 134; forest (Szentgál): 64, urban (Veszprém): 70

^4^ Number of nestlings (broods), total: 488 (84); forest (Szentgál): 215 (33), urban (Veszprém): 273 (51)

^5^ overall effect of nestling age: ANOVA F2, 78 = 1.474, *P* = 0.234

^6^ overall effect of nestling age: ANOVA F2, 78 = 0.943, *P* = 0.394

^7^ overall effect of nestling age: ANOVA F2, 78 = 12.655, *P* < 0.001

**Table S2.** The impacts of the anthropause on great tits’ reproductive success at two urban study sites (Budapest and Veszprém) that responded differently to the lockdown in 2020 (Veszprém: reduced human activity compared to earlier years, Budapest: increased human activity compared to 2019, see Fig. 1b of the main text). The reference year is 2019. The table shows the results of the full statistical models that were used for calculating the contrasts presented in Table 1b of the main text. Budapest and 2020 were set in the intercept. Results of hatching success (the proportion of hatched chicks/laid eggs) and nestling survival (the proportion of fledged young/hatched chicks) are given on the logit scale. Study site × year interactions are highlighted in bold if statistically significant (*P* < 0.05).

| **Between-cities comparisons (2019 and 2020)** | | b ± SE | t-value | *P* |
| --- | --- | --- | --- | --- |
| *Clutch size ^1^* | Intercept | 6.95 ± 0.27 | 25.37 | < 0.001 |
|  | Site (Veszprém) | 1.48 ± 0.35 | 4.27 | < 0.001 |
|  | Year (2019) | 0.43 ± 0.41 | 1.06 | 0.290 |
|  | Site × Year | -0.09 ± 0.51 | -0.19 | 0.852 |
| *Hatching success ^2^* | Intercept | 1.16 ± 0.33 | 3.54 | < 0.001 |
|  | Site (Veszprém) | 0.98 ± 0.45 | 2.19 | 0.031 |
|  | Year (2019) | 0.71 ± 0.55 | 1.28 | 0.204 |
|  | Site × Year | -1.03 ± 0.69 | -1.50 | 0.138 |
| *Number of fledglings ^3^* | Intercept | 3.14 ± 0.68 | 4.61 | < 0.001 |
|  | Site (Veszprém) | 0.86 ± 0.80 | 1.07 | 0.289 |
|  | Year (2019) | 0.39 ± 0.95 | 0.41 | 0.682 |
|  | Site × Year | -1.16 ± 1.13 | -1.02 | 0.309 |
| *Fledging success ^4^* | Intercept | 0.05 ± 0.39 | 0.12 | 0.907 |
|  | Site (Veszprém) | 0.03 ± 0.45 | 0.07 | 0.942 |
|  | Year (2019) | 0.03 ± 0.54 | 0.06 | 0.953 |
|  | Site × Year | -0.39 ± 0.63 | -0.63 | 0.531 |
| *Nestling body mass (g) ^4^* | Intercept | 12.86 ± 0.66 | 19.50 | < 0.001 |
|  | Site (Veszprém) | 0.63 ± 0.67 | 0.95 | 0.348 |
|  | Year (2019) | 2.68 ± 0.74 | 3.62 | < 0.001 |
|  | Nestling age (day 15) ^5^ | 0.45 ± 0.49 | 0.91 | 0.366 |
|  | Nestling age (day 16) ^5^ | 0.67 ± 0.59 | 1.14 | 0.258 |
|  | **Site × Year** | **-2.59 ± 0.90** | **-2.89** | **0.005** |
| *Nestling tarsus length (mm) ^4^* | Intercept | 18.07 ± 0.28 | 63.48 | < 0.001 |
|  | Site (Veszprém) | 0.47 ± 0.66 | 1.62 | 0.109 |
|  | Year (2019) | 1.35 ± 0.32 | 4.22 | < 0.001 |
|  | Nestling age (day 15) ^6^ | -0.02 ± 0.21 | -0.11 | 0.916 |
|  | Nestling age (day 16) ^6^ | 0.30 ± 0.25 | 1.20 | 0.236 |
|  | **Site × Year** | **-1.54 ± 0.39** | **-3.98** | **< 0.001** |
| *Nestling wing length (mm) ^4^* | Intercept | 38.47 ± 1.50 | 25.64 | < 0.001 |
|  | Site (Veszprém) | -0.01 ± 1.52 | -0.01 | 0.994 |
|  | Year (2019) | 5.94 ± 1.68 | 3.53 | < 0.001 |
|  | Nestling age (day 15) ^7^ | 2.46 ± 1.11 | 2.21 | 0.031 |
|  | Nestling age (day 16) ^7^ | 5.15 ± 1.34 | 3.84 | < 0.001 |
|  | **Site × Year** | **-5.76 ± 2.05** | **-2.82** | **0.006** |

^1^ Number of broods: total: 112, urban (Budapest): 40, urban (Veszprém): 72

^2^ Number of broods, total: 107; urban (Budapest): 36, urban (Veszprém): 71

^3^ Number of broods: total: 99, urban (Budapest): 29, urban (Veszprém): 70

^4^ Number of nestlings: total: 394, urban (Budapest): 121, urban (Veszprém): 273

^5^ overall effect of nestling age: ANOVA F2, 70 = 0.738, *P* = 0.482

^6^ overall effect of nestling age: ANOVA F2, 70 = 1.012, *P* = 0.369

^7^ overall effect of nestling age: ANOVA F2, 70 = 7.468, *P* = 0.001

**3. Birds’ reproductive success: forest-city (Szentgál vs. Veszprém) comparisons using long-term data on reproductive success (2013-2020)**

To complement our analyses comparing the forest (Szentgál) and city (Veszprém) study sites in 2019 and 2020 (see Table 1a in the main text and Table S1 above) we ran further analyses. First, to disentangle the impacts of the anthropause in 2020 from natural year-to-year variation, we compared the forest-city differences (Szentgál vs. Veszprém) in birds’ reproductive success in 2020 (i.e. during the lockdown) to the long-term mean differences between these two habitat types measured in the 2013-2018 reference period.

The visual inspection of the graphs on long-term breeding success (Fig. S3) indicated two important patterns. First, the graphs suggested a negative trend for most components of birds’ reproductive success throughout the study period (2013-2020) – a decline that is more prominent in forest birds. Second, that in 2019, similarly to 2020, birds in both habitats reached generally lower reproductive success than in most preceding years, suggesting very similar forest-city differences in 2019 (without lockdown) as in 2020 (with lockdown). Thus, to test this latter conjecture, we conducted additional forest-city comparison analyses similar to that described above in this section (i.e. 2020 vs. the long term mean of the 2013-2018 period), only that now we compared the forest-city difference in 2019 (instead of 2020) to the long-term mean difference between these habitats (calculated from 2013-2018).

We fitted separate linear models for each component of the reproductive success as described in the main text (Methods), except that now the habitat × year interaction term contained 2013-2020 (i.e. an eight-level factor for “year”). Then we extracted the forest-city contrasts for each year and calculated a single, overall mean describing the whole reference period (i.e. for 2013-2018) and compared this to the forest-city difference in 2020 or 2019. For further details on calculating the contrasts see Methods in the main text.

In 2020, we found smaller forest-city differences in several components of birds’ reproductive success than in the long-term reference period (2013-2018). The reductions in habitat differences were significant for clutch size, the number of fledglings, and nestling survival, whereas the changes were not significant for hatching success or any of the studied nestling body size traits (Table S3.a). Additionally, and very importantly, however, when comparing the forest-city differences in breeding success in 2019 to that of the long-term reference period (2013-2018) we found qualitatively the same results as in the 2020 vs. long-term reference period comparisons (Table S3.b). Thus, these results indicate that, although the typical and prominent differences in breeding performance between the forest and urban populations were indeed mitigated in 2020, the same reductions in the forest-city differences were also present in 2019, when there was no lockdown.

**Fig. S3.** Changes in reproductive success of great tits breeding in a forest (Szentgál) and two urban (Veszprém and Budapest) habitats during the study, including the lockdown in 2020. Results are from linear models’ estimates (mean ± SE). For hatching success (hatched chicks/laid eggs) and nestling survival (fledged young/hatched chicks) the results were back-transformed from log odds ratio scale to probability scale to aid the interpretation. For comparisons of Veszprém (urban) and Szentgál (forest) in 2020 or 2019 to the long-term reference period (2013-2018) see Table S3; for comparisons of the two urban sites, Veszprém and Budapest (2019-2020), see Table S2.


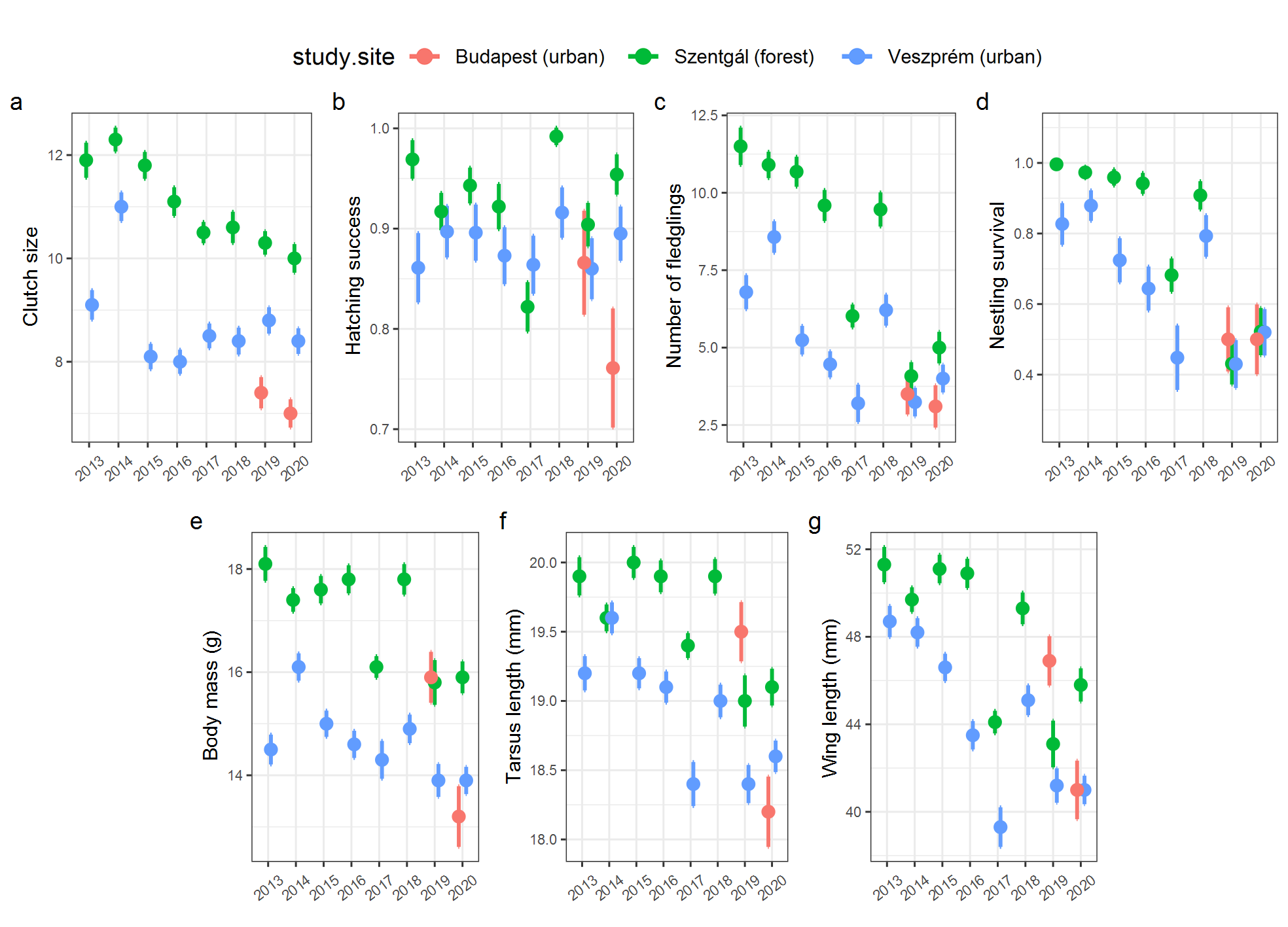


**Table S3.** Changes in population differences in the reproductive success of great tits between a forest (Szentgál) and an urban (Veszprém) habitat using long-term data. We compared forest-city differences in (a) 2020 (when breeding occurred during the lockdown) and also in (b) 2019 (with no lockdown) to a long-term reference period (2013-2018). Forest-city population differences are expressed as the forest minus urban habitat (2020 or 2019 minus the reference period), i.e. negative values indicate reduced between-habitat differences in 2020 or 2019 than in the reference period. Modell estimated contrast ± SE values are on the original scales except for hatching success (the proportion of hatched chicks/laid eggs) and nestling survival (the proportion of fledged young/hatched chicks), which are given on the log odds ratio scale. The results for nestlings’ body mass, tarsus length, and wing length are averaged over the levels of nestling age (i.e. the age when nestlings were measured post-hatch: day 14, 15, or 16). Statistically significant results (*P* < 0.05) are highlighted in bold and marginally non-significant results (0.05 < *P* < 0.1) are shown in italic.

| **Comparisons between forest and urban habitats**  (a) Habitat diff. in 2020 – habitat diff. in the reference period | contrast ± SE | t or z ratio | *P* |
| --- | --- | --- | --- |
| *Clutch size ^1^* | -0.96 ± 0.40 | -2.39 | **0.017** |
| *Hatching success ^2^* | -0.03 ± 0.60 | 0.05 | 0.962 |
| *Number of fledglings ^2^* | -2.95 ± 0.74 | -3.98 | **< 0.001** |
| *Nestling survival ^2^* | -1.96 ± 0.58 | -3.34 | **< 0.001** |
| *Nestling body mass (g) ^4, 5^* | -0.49 ± 0.44 | -1.12 | 0.261 |
| *Nestling tarsus length (mm) ^4, 5^* | -0.19 ± 0.19 | -1.01 | 0.316 |
| *Nestling wing length (mm) ^4, 5^* | 0.67 ± 1.07 | 0.63 | 0.532 |
| (b) Habitat diff. in 2019 – habitat diff. in the reference period |  |  |  |
| *Clutch size ^5^* | -0.98 ± 0.38 | -2.62 | **0.009** |
| *Hatching success ^7^* | -0.43 ± 0.44 | -0.98 | 0.325 |
| *Number of fledglings ^8^* | -3.10 ± 0.71 | -4.37 | **< 0.001** |
| *Nestling survival ^8^* | -1.96 ± 0.57 | -3.42 | **< 0.001** |
| *Nestling body mass (g) ^4, 9^* | -0.75 ± 0.56 | -1.34 | 0.181 |
| *Nestling tarsus length (mm) ^4, 9^* | -0.10 ± 0.24 | -0.43 | 0.668 |
| *Nestling wing length (mm) ^4, 9^* | *-2.28 ± 1.38* | *-1.65* | *0.099* |

^1^ Number of broods, total: 485, urban (Veszprém): 249, forest (Szentgál): 236

^2^ Number of broods, total: 466, urban (Veszprém): 241, forest (Szentgál): 225

^3^ Number of broods, total: 438, urban (Veszprém): 212, forest (Szentgál): 226

^4^ Results are averaged over the levels of nestlings’ age at ringing (three-level factor)

^5^ Number of nestlings, total: 3178; urban (Veszprém): 1264; forest (Szentgál): 1914

^6^ Number of broods, total: 497, urban (Veszprém): 247, forest (Szentgál): 250

^7^ Number of broods, total: 481, urban (Veszprém): 240, forest (Szentgál): 241

^8^ Number of broods, total: 444, urban (Veszprém): 210, forest (Szentgál): 234

^9^ Number of nestlings, total: 3046; urban (Veszprém): 1195; forest (Szentgál): 1851

**Literature cited**

Bailly, J., B. Faivre, N. Bernard, M. Sage, N. Crini, V. Driget, S. Garnier, D. Rieffel, and R. Scheifler. 2017. Multi-Element Analysis of Blood Samples in a Passerine Species: Excesses and Deficiencies of Trace Elements in an Urbanization Study. Frontiers in Ecology and Evolution 5:6.

Corsini, M., P. Marrot, and M. Szulkin. 2019. Quantifying human presence in a heterogeneous urban landscape. Behavioral Ecology 30:1632–1641.

Fenoglio, M. S., M. R. Rossetti, and M. Videla. 2020. Negative effects of urbanization on terrestrial arthropod communities: A meta-analysis. Global Ecology and Biogeography 29:1412–1429.

Halekoh, U., S. Højsgaard, and J. Yan. 2006. The R Package geepack for Generalized Estimating Equations. Journal of Statistical Software 15:1–11.

Jones, E. L., and S. R. Leather. 2012. Invertebrates in urban areas: A review. European Journal of Entomology 109:463–478.

Liang, Y., I. Rudik, E. Y. Zou, A. Johnston, A. D. Rodewald, and C. L. Kling. 2020. Conservation cobenefits from air pollution regulation: Evidence from birds. Proceedings of the National Academy of Sciences of the United States of America 117:30900–30906.

Peach, W. J., K. E. Vincent, J. a. Fowler, and P. V. Grice. 2008. Reproductive success of house sparrows along an urban gradient. Animal Conservation 11:493–503.

Salma, I., M. Vörösmarty, A. Z. Gyöngyösi, and ... 2020. What can we learn about urban air quality with regard to the first outbreak of the COVID-19 pandemic? A case study from Central Europe. Atmospheric ….

Salmón, P., E. Stroh, A. Herrera-Dueñas, M. von Post, and C. Isaksson. 2018. Oxidative stress in birds along a NOx and urbanisation gradient: An interspecific approach. Science of the Total Environment 622–623:635–643.

Sicard, P., E. Agathokleous, V. Araminiene, E. Carrari, Y. Hoshika, A. De Marco, and E. Paoletti. 2018, December 1. Should we see urban trees as effective solutions to reduce increasing ozone levels in cities? Elsevier Ltd.

Sicard, P., A. De Marco, E. Agathokleous, Z. Feng, X. Xu, E. Paoletti, J. J. D. Rodriguez, and V. Calatayud. 2020. Amplified ozone pollution in cities during the COVID-19 lockdown. Science of the Total Environment 735:139542.
